# Individual differences in musical melody perception moderate the speech-to-song illusion in Mandarin Chinese listeners

**DOI:** 10.1038/s41598-026-44268-z

**Published:** 2026-03-23

**Authors:** Tamara V. Rathcke, Massimiliano Canzi

**Affiliations:** Department of Linguistics, University of Konstanz, 78464 Konstanz, Germany

**Keywords:** Neuroscience, Psychology, Psychology

## Abstract

Repeated exposure to a spoken phrase can give rise to the perception of the *speech-to-song illusion* (STS), whereby speech gains musical qualities and begins to sound like singing. STS is known to rely on acoustic cues and may depend on an individual’s ability to extract musical qualities (such as melody and rhythm) from speech acoustics. So far, most research has examined listeners of non-tonal languages, with preliminary evidence indicating that tonal-language listeners experience STS differently, if at all. This study investigated STS in Mandarin Chinese listeners who rated song-likeness of Mandarin sentences before and after repetition and completed the Musical Ear Test. Test sentences were designed to promote the acoustic transmission of either melody or rhythm. Results demonstrated a modest STS effect in Mandarin listeners at the group level, which was independent of sentence acoustics. Individual abilities in rhythm perception had no impact on STS while, somewhat surprisingly, weaker melody perception abilities were found to facilitate STS. This suggests that STS in Mandarin Chinese may be linked to a perceptual distortion of pitch. Overall, the findings indicate that STS mechanisms are shaped by linguistic background of listeners and provide new evidence that language experience can influence music perception and cognition.

## Introduction

Research has shown that acoustically identical phrases spoken in a non-tonal language like English, French, or German can be perceived either as speech or as song by native listeners^1–5^. For these listeners, a spoken phrase transforms into singing when repeated multiple times^1–5^. This perceptual transformation has come to be widely known as “the speech-to-song illusion” (henceforth STS)^1,6^. STS was first described by Diana Deutsch^6^ who argued that the illusion was not merely a matter of interpretation, since after hearing a spoken phrase several times, listeners were able to sing it back with altered pitches that formed a coherent song melody^1^. Behavioral perception studies in non-tonal languages have provided robust evidence that certain pitch cues of spoken phrases – specifically, stable local pitch trajectories rather than dynamic pitch movements – foster and enhance the experience of STS in these phrases^3,7,8^ It has also been shown that STS is more likely to occur in phrases containing phonological structures that give rise to high-sonority profiles (i.e., acoustic signals rich in resonance and harmonics)^9^ which, in turn, facilitate the extraction of pitch information^5^. In a similar vein, neuroimaging studies have identified that repetition of speech activates brain areas linked to pitch processing (such as the right mid-posterior superior temporal sulcus and middle temporal gyrus)^1,2^ while the strength of the illusion seems to correlate with an increased activation in a left fronto-temporal network (comprising the inferior frontal gyrus, frontal pole, and temporal pole) associated with prosodic evaluation^10^. Moreover, individual perceptual sensitivity to pitch can also shape individual experience of STS^3,11^. Recent research^11^ has established that the ability to direct attention exclusively to pitch of spoken phrases is associated with an increased strength of STS, without giving rise to a general tendency to perceive speech as musical. Similarly, higher perceptual skill in tonality alignment – measured as the ability to determine whether or not a sung melody closely follows or deviates from a scalar tonal framework – is also linked with an increased strength of STS but not a broad musical bias^11^. Overall, existing studies jointly highlight a crucial role of acoustic pitch cues and perceptual pitch processing in linking linguistic and musical representations during STS.

To account for the high importance of pitch in STS, it has been proposed that the illusion arises through a mechanism of perceptual re-weighting of pitch, shifting from a “speech perception mode” to a “music perception mode” during repetitions^1,3,11–14^. According to a prominent account of the STS mechanism^1,14^, pitch is typically down-weighted during speech perception because, in non-tonal languages which have primarily been studied to date, pitch plays a less important role in conveying lexical information than spectral cues. During repetition, however, acoustic properties of pitch gain perceptual salience and may involve a perceptual distortion whereby pitches of syllables are reshaped to align with musical templates^1,14^. Recent work indicates that distortion processes during STS affect the perception of rhythm and timing rather than pitch while the perception of pitch taps into stored musical representations and benefits from rich internal repertoires of musical melodies that individual listeners might have^15^. In any case, the proposed mechanism explains STS experience exclusively in listeners whose native language is non-tonal (such as English, German, or French). In tonal languages (such as Mandarin, Cantonese, Vietnamese, Thai, or Hmong), down-weighting of pitch cannot be assumed to be part of the “speech perception mode”. On the contrary, no other language imposes a greater necessity on its listeners to attend to pitch patterns in speech and to establish perceptual frame of reference for the interpretation of these patterns than a tonal language. Tonal languages use distinct patterns of stable and/or dynamic pitch trajectories to convey and distinguish lexical meanings. In a tonal language, each syllable of every word typically has a pitch specification and, when combined into longer phrases and utterances, gives rise to complex pitch phenomena^16,17^. It has been suggested that due to their pitch variation, spoken utterances in tonal languages are melodically rich and at times resemble music^18^. Moreover, one prominent hypothesis proposes that, in contrast to non-tonal languages, contemporary tonal languages bear a closer resemblance to the shared-origin ‘musilanguage’ from which both human language and music might have evolved^19,20^.

Given the high relevance of tone within the linguistic system, it is perhaps not surprising that native experience with a tonal language may enhance pitch perception in music. Several studies have consistently documented that listeners of tonal languages outperform listeners of non-tonal languages on all tasks involving the perception of musical melodies^21–24^. It has been suggested that this superiority in pitch perception does not stem from differences in basic auditory thresholds^24,25^. Rather, the advantage likely arises from higher-level auditory processing abilities, such as enhanced sensitivity to global pitch patterns and greater working memory capacity for pitch^24,25^. It would seem that these perceptual abilities might predispose listeners of tonal languages to perceiving STS more strongly than listeners of non-tonal languages, which is, however, not the case^26^. Preliminary evidence indicates that native listeners of tonal languages have a significantly reduced experience of STS than native listeners of non-tonal languages, regardless of the language in which a spoken phrase is repeated^26^. To account for this finding, it has been suggested that tonal language speakers encode pitch patterns as linguistic due to their native language experience and are therefore less likely to reinterpret those patterns musically, which reduces their susceptibility to STS^26^. While this account aligns well with behavioral and neuroimaging evidence suggesting that tonal language speakers preferentially template pitch as linguistic^27^, it overlooks the involvement of rhythm in STS^3,11^. Indeed, an existing proposal suggests that tonal language speakers may rely more strongly on rhythmic than melodic cues when extracting the musical structure from speech and experiencing STS^8^, though evidence in support of this proposal is currently lacking. Importantly, the proposal implies that perceptual re-weighting of pitch and an accompanying shift from a “speech perception mode” to a “music perception mode” during repetitions^1,3,11–14^ cannot be assumed as a general, cross-linguistic mechanism for explaining how STS arises. This suggests that the STS mechanism is more complex than previously assumed and has to incorporate language-specific organization of pitch and rhythm in speech and song as well as listener-specific perceptual sensitivities to these dimensions. However, these factors remain poorly integrated in the current approaches to defining the STS mechanism.

Several studies indicate that in non-tonal languages, rhythmic structure of the stimulus as well as rhythm perception ability of the listener are involved in STS^3,11^. Specifically, local timing structures built around inter-vocalic intervals (in contrast to inter-syllabic intervals) appear to enhance STS, whereas global rhythmic features of spoken phrases, such as accentual regularity within a phrase or across phrase repetitions, do not significantly influence the experience^3,8,28^ In a similar vein, listeners with a stronger perceptual ability to correctly judge whether the timing of overimposed tones is synchronized with the beat of music or deviates from it tend to report a more enhanced experience of STS^11^. It has been argued that the presence of a steady beat is characteristic of music but generally absent in natural speech, and listeners who excel at beat perception may rely on this cue when experiencing repeated speech and detecting its musical structure, which, in turn, enhances their perception of the illusion^11^. However, if superior perceptual skills in beat perception facilitate STS, listeners of tonal languages are likely to be less – rather than more – susceptible to STS, given a weaker rhythm perception ability documented for listeners of tonal languages in some studies^21–24^. Compared with Japanese listeners^24^, Mandarin Chinese listeners perform significantly more poorly on the rhythm subtest of the Musical Ear Test (MET)^29^. Many Japanese dialects have a time-counting unit, the mora, which governs the duration of segments and syllables^30^, and adult Chinese learners are well known to experience particular challenges with these phonological properties of temporal structure when acquiring Japanese^31^. It appears that phonological quantity – absent in Mandarin but present in Japanese – equips listeners with enhanced rhythm perception abilities, much as linguistic experience with tone enhances perceptual abilities for musical melody^21–24^. Compared with listeners of English or other non-tonal language without phonological quantity, Mandarin Chinese listeners perform similarly on the rhythm subtest of the MET^32^. These findings are somewhat at odds with the proposal suggesting that tonal language speakers would experience STS through an enhanced perception of rhythmic, rather than tonal, structure in speech^8^. Understanding how STS may be shaped by native language and musical aptitude is crucial for advancing the understanding of the links between language experience and music perception and cognition.

While most previous research on STS has focused on listeners of non-tonal languages^1–5,28^, existing estimates indicate that the majority of languages spoken around the world are tonal^33^. This suggests that the current understanding of the mechanism and prevalence of STS may not be representative of listeners’ experience globally. The present study investigated STS in native listeners of Mandarin Chinese. Mandarin is the most prominent representative of the Sino-Tibetan language family which is the largest group of languages to use lexical tone^33^. In contrast to many African tone languages with a two-way contrast between a high and a low level tone, the tone system of Mandarin is relatively complex: it comprises one neutral tone and four lexical tones, contrasting not only properties of pitch height (five levels within a speaker’s voice range)^34^ but also pitch dynamics (level, rising, falling)^17,33^. It additionally involves a series of tone sandhi processes which modify pitch representations of the four lexical tones depending on the tonal context created by preceding and following syllables^17,33^. As a consequence of this complexity, auditory representations in Mandarin listeners may be extremely rich, incorporating detailed local and distal specifications of pitch^35^. In contrast to Cantonese (another prominent language under the umbrella of present-day Chinese), Mandarin has fewer tones, but a more distinct and stable tone system while Cantonese tones are frequently produced with a substantial pitch overlap^36^, causing tonal misperceptions^37^, potentially destabilizing the tone system and leading to sound changes in the number and acoustic properties of tone contrasts^38,39^. At the same time, congenital amusia exists among Mandarin Chinese listeners^40^, suggesting that native experience with lexical pitch in a stable and robust tone system does not deterministically shape individual ability in musical pitch perception.

The present study asked whether the perception of STS is moderated by individual sensitivity to musical melody and rhythm in Mandarin Chinese listeners, and whether individual susceptibility to STS is shaped by the acoustics of speech in a way similar to what has been observed in listeners of non-tonal languages^5^. Even though there is preliminary evidence on a reduced susceptibility of tonal-language listeners to STS^26^, existing findings are based on a very small sample (five Mandarin and five Thai listeners) and a single sentence per native language. The present study aimed to provide a more representative assessment by including a larger listener cohort, a broader set of sentence stimuli varied in rhythmic and melodic properties, and individual measures of listeners’ rhythm and melody abilities, allowing for a more nuanced examination of factors shaping STS perception in a tonal language.

We employed the MET^29^ to test musical aptitude as it offers a well-established and normed assessment of individual abilities in both melody and rhythm perception, is freely accessible, computer-administered, and fixed in duration (20 minutes), ensuring identical testing situation for all participants^32^. It has previously been used to examine musical aptitude in tonal and non-tonal-language listeners^24,29^ and was normed with a large sample of native listeners of tonal and non-tonal languages^32^. Linguistic materials consisted of two types of sentences: high-sonority and low-sonority sentences^5^. Sonority is typically conceptualized as a continuum, ranging from high (as in vowels) to low (as in voiceless stops), largely reflecting the presence of vocal fold vibration and the extent of vocal tract openness during speech production^9^. Based on these properties, high-sonority sentences promote the transmission and extraction of pitch information in speech^5^ while low-sonority sentences may increase rhythmicity, by virtue of regularizing sequences of opening and closing articulatory gestures^41^, leading to a more regular modulation of the amplitude envelope, which has been suggested to influence the perception of rhythm^42,43^. Moreover, sounds with a more regular modulation of the amplitude envelope tend to be perceived as music rather than as speech^44^. Assuming that the mechanism of STS is perceptual in nature^1–3^, we tested the hypothesis that tonal language speakers experience STS through the extraction of rhythmic rather than melodic cues to the musical structure inherent to speech^8^. We further hypothesized that high sensitivity to musical rhythm (as measured by the rhythm subtest of the MET) would equip listeners with a heightened ability to extract rhythmic structure of speech, thus leading to an enhanced experience of STS^11^. Moreover, we expected low-sonority phrases to enhance the perception of rhythmicity in speech and to lead to a higher susceptibility to the illusion in these listeners. To test these hypotheses, we created twelve phrases contrasting in phonological sonority and resulting acoustic features. We recruited eighty-four volunteers whose native language was Mandarin Chinese. The listeners first rated the song-likeness of test sentences played back to them in isolation, then completed the MET, and finally performed the STS test, listening to eight repetitions of each test sentence and rating its song-likeness after repetitions. We analyzed the song-likeness ratings before vs. after repeated exposure to high-sonority (i.e., highly melodic) sentences vs. low-sonority (i.e., highly rhythmic) sentences and examined the relationship between listeners’ ability to perceive musical melody or rhythm and their perception of STS.

## Results

### Musical Ear Test in Mandarin Chinese listeners

**Fig. 1:**
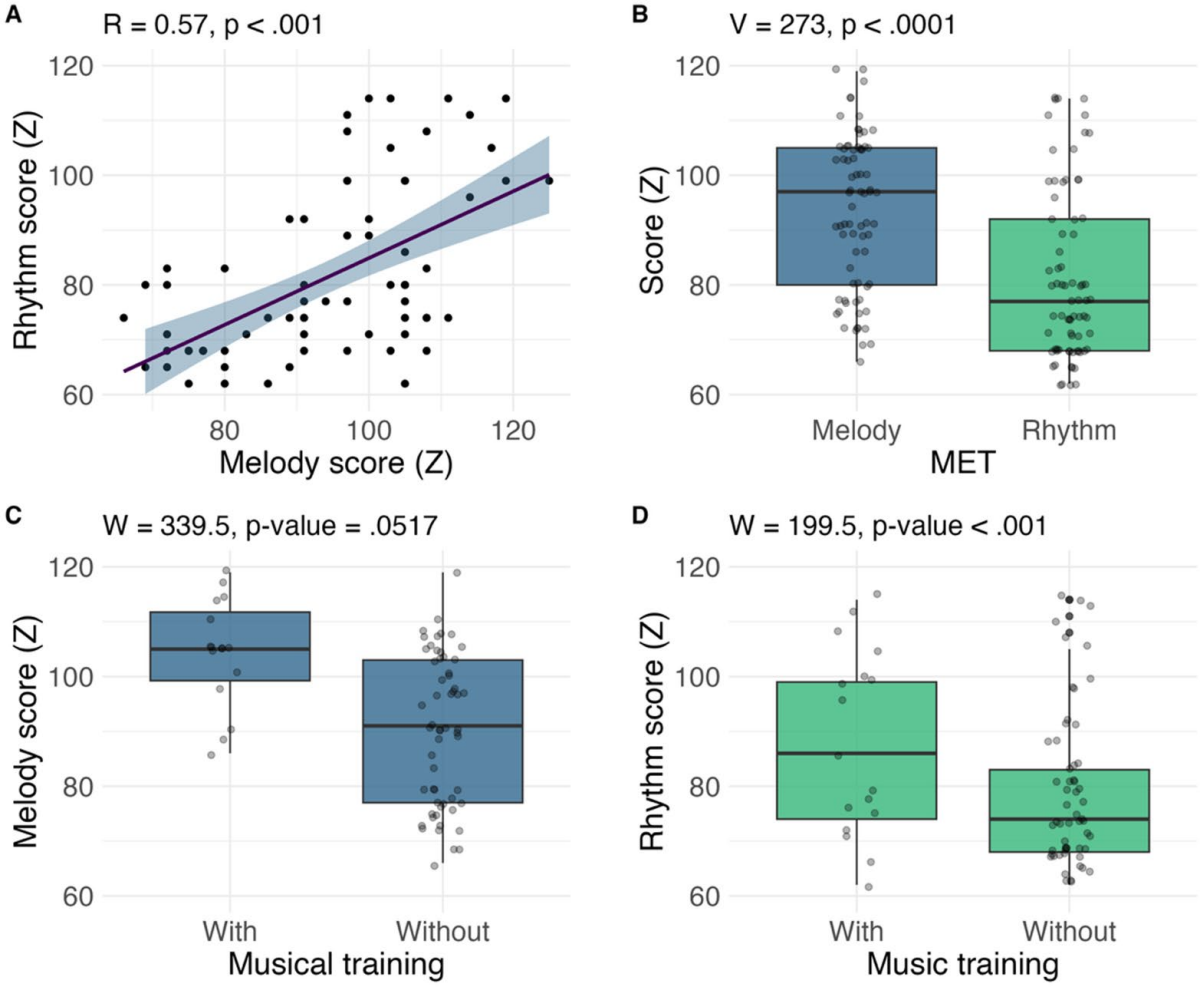
Results of the group performance on the two subtests of the Musical Ear Test, showing (**A**) a positive correlation between individual ability in the perception of musical melody and rhythm, (**B**) discrepancy between performance in the melody vs. rhythm subtest, (**C**) difference in the melody test performance between participants with and without musical training, and (**D**) difference in the rhythm test performance between participants with and without musical training.

We first tested for covariance between the two measures of the MET, the ability to identify changes in rhythm vs. melody when auditorily comparing two short musical phrases^29^. We found a strong positive correlation between the two scores (R^2^ = 0.57, *p* < .001, see Fig. 1-A), indicating that higher perceptual sensitivity to rhythm fluctuations typically went hand in hand with higher perceptual sensitivity to fluctuations in musical melody^32^. An additional test examined if performance on the melody and rhythm subtests of the MET was comparable or different among the participants of the present study^24,32^. Wilcoxon signed rank test with continuity correction demonstrated a significant discrepancy between the two scores, confirming that the Mandarin participants of the present study tended to score significantly higher on the melody than the rhythm subtest (V = 273, *p* < .001, see Fig. 1-B)^24,32^. Overall, these results were in alignment with previous studies of musical aptitude in tonal-language listeners, tested using the MET^24,29,32^. Musically trained participants had slightly higher scores on both the melody subtest (105.5 vs. 90.2, Fig. 1-C) and the rhythm subtest (87.9 vs. 78.9, Fig. 1-D) of the MET. However, Mann Whitney U-test for unpaired samples showed a significant difference between the two groups of participants only for the rhythm subtest (W = 199.5, *p* < .001), possibly due to a relatively low number of musically trained participants (*n* = 17) and a large overlap in scores between the two groups (see Fig. 1-C and D). The raw Cronbach’s alpha was 0.73 for the melody score, and 0.63 for the rhythm score.

## Evidence for a musical bias

We then examined if a musical bias could be observed in the data. Musical bias was defined the tendency to perceive and rate all sentences as more song-like before experiencing repetitions (i.e., at baseline). There was no evidence that individual variability in either musical melody (F(1,73) = 2.083, *p* = .15) or rhythm perception (F(1,73) = 0.001, *p* = .97) influenced the baseline ratings of test sentences. That is, there was no evidence for a musical bias arising from higher musical aptitude^11^.

## Evidence for STS

**Fig. 2:**
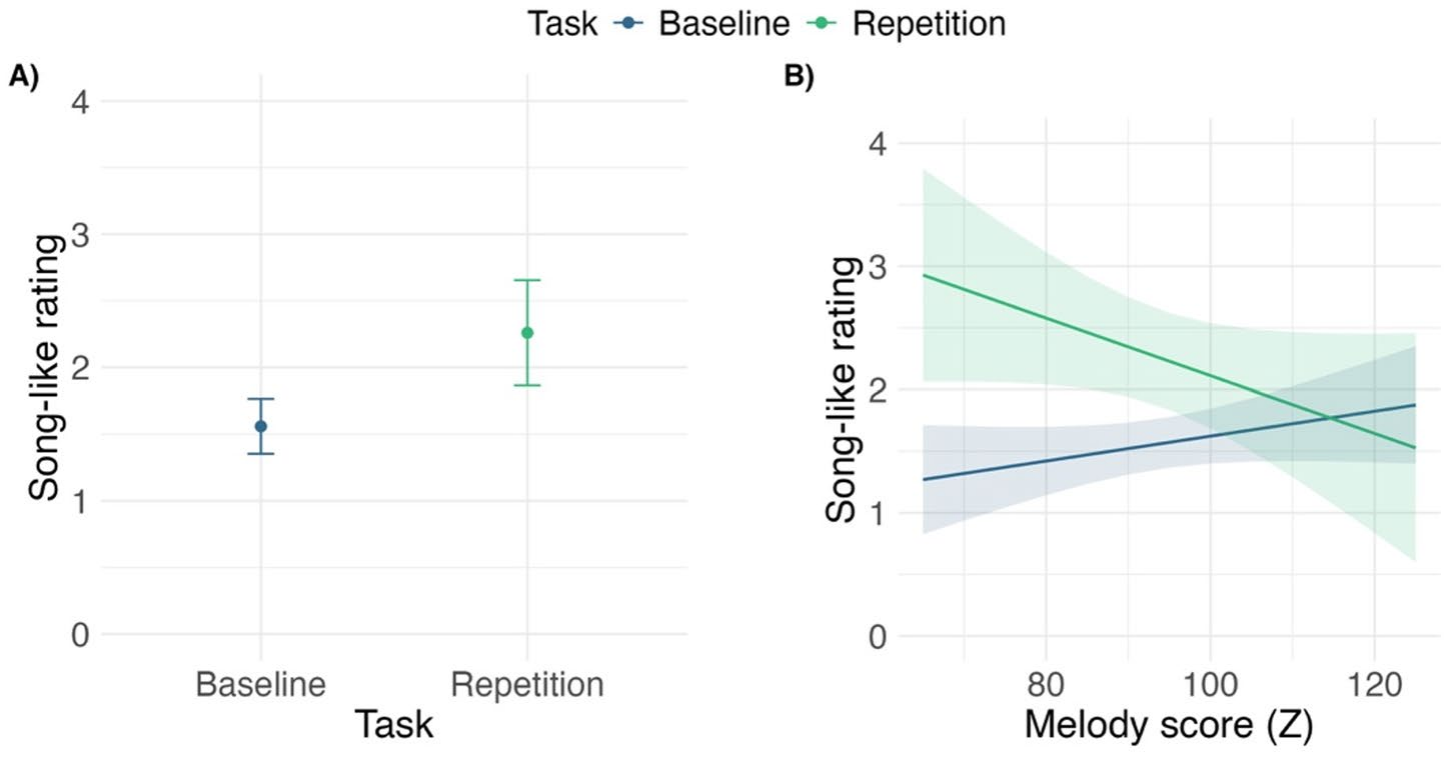
(**A**) Model estimates obtained from a model comparing Mandarin listeners’ perception of song-likeness of test sentences in the baseline task (i.e., before exposure to sentence repetitions) vs. after exposure to sentence repetitions. (**B**) Model estimates obtained from a model comparing song-likeness perceptions of test sentences before (baseline) vs. after exposure to sentence repetitions in listeners with different levels of individual melody perception ability. Song-like ratings were collected on an 8-point scale from 1 = *clearly speech* to 8 = *clearly song*.

Next, we tested if exposure to sentence repetitions led to an STS experience in Mandarin listeners of the present study. The results showed that there was a slight but significant increase in song-likeness ratings of all test sentences after repetitions (F(1,73) = 13.85, *p* < .001, see Fig. 2-A) as compared to song-likeness ratings at baseline (ß = 0.70, SE = 0.18, df = 73.0, t = 3.87, *p* < .001), though there was no indication that the increase in song-likeness was influenced by the sonority of test sentences (F(1,1637) = 0.0054, *p* = .94).

## Individual factors of an STS experience

Lastly, we tested the hypotheses of the present study by examining the role of musical aptitude in STS. The best-fit model retained an interaction of the task (baseline vs. repetition) and individual melody perception (F(1,73) = 7.08, *p* < .01, see Fig. 2-B and Table 1) but not individual rhythm perception (F(1,73) = 1.82, *p* = .18, see Table 2). There was also no evidence for an interaction of sentence sonority and individual rhythm perception ability in song-likeness ratings received for the test sentences after repetitions (F(1,812) = 0.07, *p* = .80), refuting the main hypothesis of the study that tonal-language speakers would experience STS through an enhanced perception of rhythmic rather than tonal structure in speech^8^. By-sentence variability of random effects was relatively low in these data (SD = 0.10). In contrast, the best-fit model revealed more substantial between-listener variability: the standard deviation of participant random intercepts (SD = 0.83) was indicative of individual differences in baseline ratings whereas the standard deviation of participant-specific random slopes for the task (baseline vs. repetition, SD = 1.52) suggested rather marked heterogeneity in listeners’ susceptibility to STS. To understand the nature of the interaction between the task and individual melody perception (Fig. 2-B), we ran a post-hoc analysis using the *emmeans()* function and comparing the difference in song-likeness responses at baseline vs. after repetitions in listeners who scored in the first (80) vs. third (105) quartile in the melody subtest of the MET. Bonferroni correction for multiple comparisons was applied. The results of the post-hoc analysis showed that only those listeners who scored low on the melody perception in the MET reported significantly higher song-likeness experience for spoken sentences after repetition (ß = 1.16, SE = 0.25, df = 73.0, t = 4.64, *p* < .001). In contrast, listeners who scored high on the melody perception did not report a significant increase in sentences’ song-likeness after repetitions compared to the song-likeness at baseline (ß = 0.32, SE = 0.23, df = 73.0, t = 1.40, *p* = 1.00), indicating that these Mandarin listeners did not experience STS.

**Table 1:** Likelihood ratio tests for the three-way interaction of condition (high vs. low sonority), task (baseline vs. repetition), and individual melody scores. Note: β, standard deviation (sd), and χ² values are rounded to two decimals for readability.

Term	β	sd	χ^2^	df	*p*
intercept	0.49	0.70	0.50	1	0.481
condition (low)	0.25	0.41	0.36	1	0.547
task (repetition)	4.17	1.23	11.57	1	< 0.001
melody	0.01	0.01	2.60	1	0.107
condition × task	–0.68	0.58	1.35	1	0.245
condition × melody	–0.01	0.01	0.62	1	0.429
task × melody	–0.04	0.01	8.19	1	< 0.01
condition × task × melody	0.01	0.01	1.36	1	0.244

**Table 2:** Likelihood ratio tests for the three-way interaction of condition (high vs. low sonority), task (baseline vs. repetition), and individual rhythm scores. Note: β, standard deviation (sd), and χ² values are rounded to two decimals for readability.

Term	β	sd	χ^2^	df	*p*
intercept	1.71	0.65	8.85	1	0.003
condition (low)	2.18	1.17	0.50	1	0.481
task (repetition)	–0.01	0.02	3.75	1	0.053
rhythm	–0.26	0.38	0.03	1	0.857
condition × task	–0.05	0.04	0.02	1	0.876
condition × rhythm	0.08	0.54	0.26	1	0.611
task × rhythm	0.01	0.01	1.62	1	0.203
condition × task × rhythm	–0.01	0.02	0.03	1	0.863

## Discussion

The present study investigated the perception of STS in Mandarin Chinese listeners – native speakers of a tonal language who are known to be less susceptible to the illusion as compared to speakers of a non-tonal native language^26^. Here, we examined a previously proposed mechanism of STS for tonal listeners^8^ and focused on the question whether or not musical aptitude moderated individual experience of STS and how pitch and rhythm structure of spoken phrases influenced STS in these listeners. Specifically, the study aimed to empirically examine the proposed mechanism that tonal-language listeners perceived the transformation from speech to song through a perceptual re-evaluation of rhythmic, rather than melodic, cues as musical during repetitions^8^. Building on this proposal, participants completed the rhythm and melody subtests of the Musical Ear Test^29,32^ and rated a series of sentences spoken in their native language on the 8-point song-likeness scale before and after listening to sentence repetitions. The test sentences contrasted in phonological sonority, with high-sonority sentences promoting the acoustic transmission of pitch and melody^5^ and low-sonority sentences promoting the transmission of rhythmicity in speech^42,43^. We hypothesized that listeners with high beat perception ability would experience the illusion more vividly, especially in low-sonority (i.e., more rhythmic) phrases. Overall, the present study showed a rather modest STS effect in Mandarin listeners (as, e.g., compared to findings of STS in listeners with a non-tonal native language^3,5,7,8,45^, thus confirming previous observations that speaking a tonal language natively may reduce individual susceptibility to STS^26^. Moreover, the results did not provide support for the main hypothesis of the present study: there was no effect of either sentence sonority or beat perception ability, suggesting that the current hypothesis about the perceptual mechanisms underpinning the STS experience in listeners of tonal languages^8^ requires revision. Based on the present findings, it appears very unlikely that tonal-language listeners rely more strongly on rhythmic, rather than melodic, cues when extracting the musical structure from speech and experiencing STS^8^. Instead, the strength of perceptual encoding of pitch and melody may be at play when native speakers of a tonal language perceive the transformation from speech to song^26^. We found that those Mandarin listeners who had a somewhat more vivid song-likeness experience of spoken sentences after repetition were the listeners who scored relatively low on the melody perception subtest of the MET. In contrast, high individual ability in melody perception completely blocked the STS experience in Mandarin listeners of the present study. This finding is noteworthy for several reasons. First and foremost, it supports the idea that the perception of pitch and melody may be a major – perhaps universal – key to the STS experience across listeners of different native languages^3,5,11,26^, though the exact role of pitch perception ability appears to depend on listeners’ native language. In non-tonal languages like English or German, superior pitch perception abilities promote STS, indicating that the illusion is shaped by individual ability to detect and extract musical structure in speech^3,11,15^. However, in tonal languages (or at least in Mandarin Chinese), the opposite effect is found: higher pitch perception ability hinders STS. This finding resonates with an idea that goes back to Diana Deutsch’s original discovery of STS, when she proposed that some form of perceptual distortion must occur during repetitions for spoken pitch to be experienced as sung melody^1^. Her original account that pitch contours of spoken language are perceptually altered so they conform to a well-formed melody aimed at explaining STS in non-tonal languages while our results point to such distortion being involved in the STS perception among Mandarin listeners. Following this idea^1^, we suggest that weaker encoding of melody in Mandarin listeners may cause them to release veridical representations of linguistic pitches, which enables somewhat distorted, more song-like representations of musical melody to arise in listeners’ minds during sentence repetitions. In contrast, listeners with stronger melody perception ability who encode pitch more faithfully, seem to be reluctant to relinquish perceptual representations of lexical tone, might not experience a perceptual distortion of pitches, and thus do not experience the transformation from speech to song. Importantly, songs sung in Mandarin Chinese share several relevant acoustic features with songs performed in non-tonal languages (viz. stable, sustained pitch)^46^, suggesting that the results of a perceptual distortion by Mandarin listeners and a perceptual melody extraction by non-tonal-language listeners may largely overlap. Behavioral paradigms which require participants to produce sentences as they hear them after repetitions^1^ would be best suited to examine if this hypothesis is correct.

While it is commonly believed that songs composed in tonal languages obey the “text-setting constraint”^47^, holding that song melody ought to be aligned with lexical tones of the language to preserve the intelligibility of song lyrics, tonal languages can greatly vary with regards to their implementation of this constraint^48^. Mandarin popular music, in particular, largely disregards it, prioritizing musical melody over lexical tone^46,49^. In a majority of Mandarin songs, tone and melody are misaligned, as neither contours nor transitions of lexical tones are consistently mirrored in music^48–50^. In contrast, Cantonese singers tend to follow lexical tone more closely and even reintroduce tonal contours if they are missing in musical melody^51^. Overall, Mandarin singers appear to suppress lexical tone information in musical contexts, whereas Cantonese singers retain it^46,48,49,51,52^. It has been argued that differences in linguistic characteristics (such as the ratio of monosyllabic to polysyllabic words, homophone density, and the functional load of tone) between Cantonese and Mandarin may contribute to the contrasting degrees of tone-melody correspondence observed in their respective popular music traditions^52^. Historically, the influence of lexical tone on Chinese song composition was observed to vary among individual composers and to be more strongly aligned with a conservative version of older Chinese and its music (rather than with contemporary, low-prestige dialects of Chinese)^34^.

The contrast between Mandarin and Cantonese singers implies that daily music experience^53^ is likely to differ among speakers of different tonal languages and that such speakers cannot be treated as a homogenous group when it comes to the understanding of the role that lexical tone plays in the perception of STS. As previously argued^4^, a hypothetical switch from a linguistic to a musical listening mode during STS is too simplistic a mechanism to account for the complexities of individual STS experience. Instead, STS may be best understood as a perceptual illusion that emerges from integrating auditory input with prior experience and context-driven expectations^54^. We suggest that repeated exposure to a spoken phrase, required for STS to arise, establishes a context typical of music^12,53,55^ but is rare and ambiguous in language^56^ and thus creates a context that biases auditory perception toward song. This bias alone may be insufficient for STS among listeners of different native languages and requires further reinforcement by stimulus features and/or individual listener traits. The present study provides evidence for individual listener traits in Mandarin Chinese but does not clarify stimulus features which can create a strong musical bias for Mandarin listeners as these listeners seem to be very well equipped to extract pitch information from both high-sonority and low-sonority sentences tested in this study^57^. Instead, future research could examine Mandarin listeners’ perception of STS in sentences with more stable, sustained pitch^46^(e.g., contrasting sentences containing predominantly dynamic tones with sentences containing predominantly level tones), to deepen the understanding of acoustic stimulus properties that can create and reinforce musical bias in Mandarin listeners.

Acquiring a tonal language requires attention to be directed to pitch differences and pitch changes, as these signal semantic distinctions between words. This linguistically motivated perceptual sensitivity which develops from early infancy^58,59^ appears to strengthen the representation and discrimination of pitch in general, even outside of linguistic contexts. It is, however, noteworthy that despite the general tendency to excel at melody perception^21–25^, tonal-language listeners also show substantial, non-clinical individual variability in this skill as indicated in the sample of the present study. The MET used here and in previous studies^24,32^ was developed to provide a standardized and normed tool of assessing musical aptitude in musicians as well as non-musicians, capturing a wide range of abilities without producing ceiling effects^29,32^. At the same time, both subtests of the MET measure not only the perceptual ability to encode and distinguish musical rhythms and melodies, they also tap auditory working memory capacity (as measured by the Digit Span Forward test) since they require listeners to maintain auditory representations of two musical phrases before making a same-different judgment of rhythm or melody^29,32^. Working memory capacity is also known to play a role in the STS experience of non-tonal-language listeners^4^, which suggests that capacity-limited cognitive resources may have their own contribution to shaping STS, possibly in interaction with musical aptitude traits. The conclusions of the present study are therefore limited to one specific aspect of musical melody perception – the ability to encode and maintain veridical representations of musical pitch patterns in auditory working memory. It would be worthwhile replicating the results of this study by means of other tests of melody perception, less taxing to auditory working memory. For example, the mistuning perception test^60^ which evaluates listeners’ sensitivity to disruptions in song melodies and their ability to detect vocal mistuning may be a promising alternative to the MET. It tests not only the acuity in encoding pitch within a melodic contour but also the sensitivity to culturally learned norms of scale tuning in songs and singing.

Even though the present study did not reveal an effect of rhythm-related perceptual abilities on STS in Mandarin listeners, it would be premature to conclude that rhythm plays no role in creating the STS effect in tonal-language listeners. The present study focused exclusively on one perceptual aspect of STS: the strength of the illusion (measured by the song-likeness rating of phrases after repetitions). While this is an important and much-researched aspect of individual STS experience^2,7,8,11,45^, our previous work shows that it is insufficient to capture the full complexity of the phenomenon, which also encompasses the likelihood and the speed of the illusion^4,5,15^. Whether or not a listener can experience STS at all, how easily and readily the transformation occurs for them, and how vividly song-like they experience a spoken phrase after repetitions are partly dissociable aspects of the STS experience^4^. Accordingly, individual musical-aptitude profiles (as well as the acoustic-prosodic properties of spoken phrases) may influence some aspects of the illusion but not others. Evidence from a recent study with native English listeners shows that all listeners, regardless of musical aptitude, can experience the illusion, and that certain acoustic characteristics (such as stable pitch and extended periods of high sonority) make spoken phrases more likely to transform into song^15^. Musical aptitude nonetheless modulates the speed and the strength of the illusion: English listeners with weaker beat-perception abilities perceive STS more quickly, consistent with the idea of an involvement of temporal distortion processes during repetition, whereas listeners with stronger melody-perception abilities report a more vivid song-like quality of the phrase after repetitions, pointing to the extraction of musical pitch rather than pitch distortion. It is therefore possible that rhythm-related perceptual abilities in Mandarin listeners affect the likelihood and/or the speed of the illusion which were not examined in the present study. This would merit further investigation in future research on the role of rhythm in STS among tonal-language listeners^8^.

To conclude, the present study offers novel evidence that repetitions of spoken phrases may evoke the speech-to-song illusion among Mandarin Chinese listeners, particularly if these listeners have weaker melody perception ability. However, it is unclear if this finding generalizes to native speakers of other tonal languages. Since cross-linguistic, comparative research on STS is still rather scarce, future studies would benefit from including a wider variety of languages to determine potential effects of linguistic and cultural experiences on the perception of STS and, more generally, music and musical qualities in speech. Overall, our results indicate that the vividness of the song-likeness experience after repetitions may not be very strong in Mandarin Chinese listeners,^26^ and provide new evidence that language experience can influence music perception and cognition.

### Methods

#### Participants

Eighty-four native speakers of Mandarin Chinese (mean age = 23.9, range = 18–42, 54 females, 1 non-disclosed gender) volunteered to take part in the experiment running online. The participants were recruited from the Hong Kong metropolitan area via experimenters’ private networks. There was no remuneration for participation, but participants were given the opportunity to enter a voluntary prize draw upon completing all experimental tasks by providing their email address. Two winners were randomly selected from the registered email addresses and each received an Amazon voucher worth €15.

All participants confirmed that their native language was Mandarin and they did not play music professionally and had never been formally diagnosed with speech or language impairments and/or amusia, which served as exclusion criteria. Following quality checks (see below), data from a total of 75 participants (mean age = 23.6, range = 18–41, 50 females, 1 non-disclosed gender) were retained for analysis. Of these participants, only 17 (i.e., only 23% of the sample) reported having received musical training, averaging around 4 years (ranging from half a year to 12 years of formal training). Sixty-six participants reported to speak a second language, forty-five of them reported English, one person reported Japanese, one person English and German. The remaining eighteen participants did not specify which second language they spoke. None of the participants reported Cantonese as their second language, though it could not be entirely ruled out that some participants may have had exposure to Cantonese (which is widely spoken in the Hong Kong metropolitan area)^61^.

Since the present study addressed a novel research question and specifically targeted potential interaction effects, it was not feasible to estimate a plausible effect size for interaction terms in advance. Any a-priori power analysis based on speculative or unsupported assumptions would therefore have been uninformative^62^. However, the sample of the present study exceeds the sample of a previous study on STS in listeners of tonal languages (*n* = 10)^26^.

## Materials

For the STS illusion task, twelve Mandarin sentences were created (see Table 3), following the guidelines established in a previous study^5^. Six sentences were created with a high-sonority profile (i.e., they contained vowels and voiced consonants such as nasals and approximants that promote pitch transmission) and six had low-sonority (i.e., they contained many voiceless stops and fricatives that disrupt pitch transmission but increase rhythmic modulations of the amplitude envelope)^27^. The two types of sentences were matched in the total number of syllables (varying from 4 to 14 syllables in total). While the number of sentences is relatively limited (six per condition), potentially increasing susceptibility to item-specific effects, it nonetheless far exceeds the number of sentences used in previous research on STS in listeners of tonal languages (*n* = 1)^26^.

**Table 3:** Overview of the study materials.

Stimulus		1	2	3	4	5	6
High-sonority	Orthography	你们来了。	玛莉是爱尔兰人。	我爷爷养了一条鱼。	奶奶明年去南美旅游。	我姥姥的落院里很热闹。	妈妈明明忘了买柠檬、奶油和牛奶。
	IPA transcription	/ni mən laɪ lɤ/	/ma li ʂɨ aɪ ɚ lan ɻən/	/wo jɛ jɛ jɑŋ lɤ jɪ tʰjɑʊ jy/	/naɪ naɪ miŋ niæn tɕʰy nan meɪ ly jou/	/wo lɑʊ lɑʊ tɤ luo juan li xən ɻɤ nɑʊ/	/ma ma miŋ miŋ waŋ lɤ maɪ niŋ mɤŋ naɪ jou xɤ niou naɪ/
	English translation	You are here.	Mary is Irish.	My grandfather has a fish.	Grandma is traveling to South America next year.	My grandma’s courtyard is very crowded.	Mom obviously forgot to buy lemon, cream, and milk.
Low-sonority	Orthography	他在洗澡。	康太太不在厨房。	我看到橙色的柜台。	观众都支持这个看法。	他把卡片都装在口袋里。	爸爸刚刚在桃树下吃香蕉和葡萄。
	IPA transcription	/tʰa tsaɪ ɕi tsɑʊ/	/kʰɑŋ tʰaɪ tʰaɪ pu tsaɪ ʈʂʰu fɑŋ/	/wo kʰan tɑʊ ʈʂʰɤŋ sɤ tɤ kueɪ tʰaɪ/	/kuan ʈʂʊŋ təʊ ʈʂɭ ʈʂʰɭ ʈʂɤ kɤ kʰan fa/	/tʰa pa kʰa pʰiæn təʊ ʈʂuɑŋ tsaɪ kʰəʊ taɪ li/	/pa pa kaŋ kaŋ tsai tʰaʊ ʂu ɕia ʈʂʰɭ, ɕiaŋ tɕiɑʊ xɤ pʰu tʰɑʊ/
	English translation	He is in the shower.	Mrs. Kang is not in the kitchen.	I see an orange counter.	The audience supports this view.	He put all the cards in his pocket.	Dad just ate bananas and grapes under the peach tree.
Number of syllables		4	7	8	9	10	14

To verify the effectiveness of the manipulation, we computed a mean sonority index for each sentence. For this, each phoneme was assigned a numerical value on the phonological sonority scale, ranging from 0 (voiceless plosives) to 9 (open vowels)^5,9^. The mean sonority index was calculated as the average value across all phonemes in a sentence. In addition, we measured the primary acoustic-phonetic correlate of phonological sonority, the proportion of voiced, sonorous sounds in each sentence (measured as a percentage of total sentence duration, % sonorous). Welch two-sample t-tests confirmed the success of the manipulation (see Table 4). On average, sounds capable of carrying pitch information accounted for only 60% of the total duration in low-sonority sentences, but about 80% in high-sonority sentences. For alternative ways of quantifying sonority of speech signals and capturing its perceptual correlates see^63^.

**Fig. 3:**
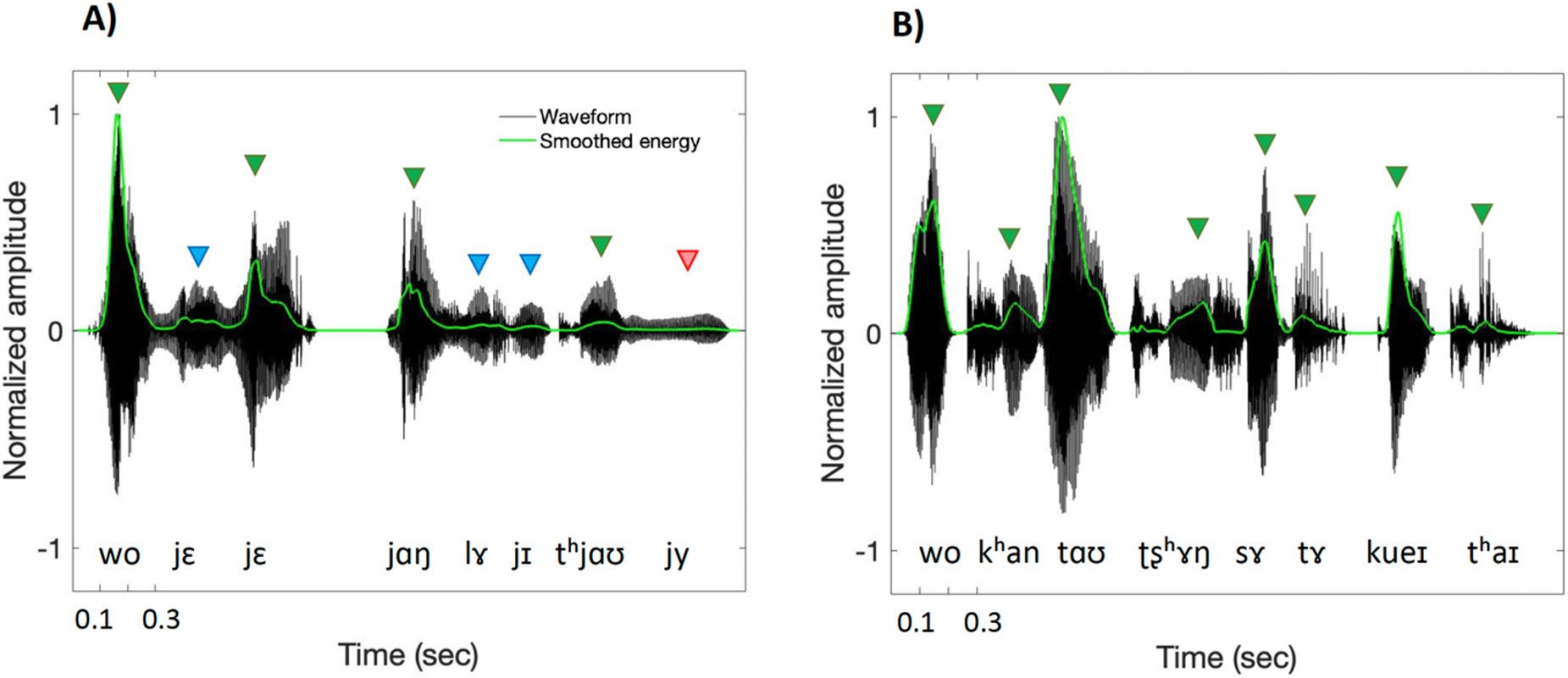
(**A**) Waveform (black lines) and smoothed energy contour (green lines) for the high-sonority sentence consisting of eight syllables: /wo jɛ jɛ jɑŋ lɤ jɪ tʰjɑʊ jy/ (*“My grandfather has a fish”*). (**B**) Waveform (black lines) and smoothed energy contour (green lines) for the low-sonority sentence consisting of eight syllables: /wo kʰan tɑʊ ʈʂʰɤŋ sɤ tɤ kueɪ tʰaɪ/ (*“I see an orange counter”*). Green triangles highlight syllables with clear amplitude modulation around a high-energy nucleus (vowel), blue triangles indicate syllables with reduced amplitude modulation, and red triangles indicate syllables that completely lack amplitude modulation.

To control for other factors influencing STS, we compared speech rate (in syllables per second) and pitch variability (in semitones) between high- and low-sonority sentences. To minimize micro-prosodic F₀ effects from intervening consonants and to ensure comparability across sonority conditions, pitch variability was calculated based on changes within vowels^5^. Specifically, F₀ was measured at 25% and 75% of each vowel’s duration, and the difference between these two points was converted into semitones. Neither speech rate nor pitch variability differed systematically and significantly between the two experimental conditions (see Table 4). We additionally conducted an analysis of the amplitude envelope^42,43^ and derived a measure of temporal amplitude variability in high- vs. low-sonority sentences. For this, we calculated smoothed energy contours for all sentenced following previously developed procedures^64–66^. The calculation of energy was averaged across successive 40-ms timeframes with a 44-sample shift and the 6th order moving-average filter, downplaying samples with zero-crossing rates exceeding 4000 (i.e., values typically associated with frication noise in the signal)^65^. The result of the calculation is showin in Fig. 3, comparing smoothed energy contour of a high- vs. low-sonority sentence with the same number of syllables. The waveform of low-sonority sentences appeared more rhythmic, due to higher temporal regularity and more pronounced peaks of syllabic amplitude modulations. We manually identified peaks and troughs in smoothed energy contours and, based on these timepoints (see arrows in Fig. 3), we calculated durations (in ms) and coefficients of variability of inter-trough intervals for all sentences. We also calculated mean syllabic energy change (in dB), by subtracting the lowest energy value at syllable onset from the highest energy value around the vowel onset. Welch two-sample t-tests on the resulting measurements confirmed that, even though amplitude modulations occurred at comparable time intervals, their variability was significantly higher in high-sonority than low-sonority sentences (see Table 4). In addition, low-sonority sentences had significantly more pronounced energy rise within syllables (cf. Figure 3-B).

All stimuli were looped with eight repetitions, each separated by a 400 ms pause^3–5^. As shown in previous research, closely spaced repetitions tend to increase the likelihood of STS perception^3^.

**Table 4:** Comparisons of phonological and phonetic characteristics of the test sentences across the two experimental conditions.

Sentence characteristics	High-sonority (mean)	Low-sonority (mean)	Results of t-tests
Sonority index	6.21	4.54	t(9.6) = 8.4, *p* < .001
% Sonorous	81.63	60.02	t(9.1) = 3.2, *p* = .01
Speech rate (syll/sec)	3.47	3.96	t(5.98) = -1.7, *p* = .14
Pitch variability (st)	1.81	1.79	t(7.3) = 0.1, *p* = .96
Mean syllabic energy rise (dB)	8.09	15.48	t(7.0) = 5.2, *p* < .01
Mean amplitude inter-trough interval (ms)	274.40	260.92	t(7.9) = 1.3, *p* = .24
Variability of amplitude inter-trough intervals (CV)	0.32	0.19	t(5.5) = 2.8, *p* < .05

### Tasks and procedure

The experiment comprised three experimental tasks (baseline test, MET, STS test) and was hosted on the online platform Gorilla^67^. At the outset, participants were presented with an information sheet about the study’s purpose, procedures, and their rights, including the option to withdraw at any time. After providing their consent to take part in this research, participants completed a questionnaire about their demographic and musical background and then proceeded to the experimental tasks. Each task was introduced by written instructions and a brief practice session, allowing participants to familiarize themselves with the interface and procedure. Participants were instructed to complete the experiment in a single session and to respond to all trials as quickly as possible. The experiment started with the baseline test in which participants listened to individual test sentences, each played only once, and rated them on a scale from (1 labeled “clearly speech”, 8 "clearly song"). These ratings served as the baseline measure of each sentence’s perceived song-likeness prior to repeated exposure^3,8,45,55^.

Subsequently, participants completed the MET^29,32^, first the melody and then the rhythm subtest. We used the English version, which is freely available from the authors^29^, and translated the test instructions into Mandarin. The MET entailed 104 trials in total, 52 per subtest. On each trial, participants had to listen to two short musical phrases and decide whether they were identical or not. Before starting each subtest, they completed two practice trials with feedback. Half of the experimental trials (26 per subtest) presented participants with identical phrases (i.e., the correct response was “same”) and the other half had different phrases (i.e., the correct response was “different”), with trial order randomized in both sessions. No feedback was given during the subtests. Phrases for the melody subtest consisted of 3–8 tones, played at a tempo of 100 bpm on a sampled piano. “Different” trials included a single pitch violation; in half of these cases (13 trials), the violation also altered the melodic contour. Twenty-five trials contained non-diatonic tones. Of the remaining 27 trials, 20 were in a major key and 7 in a minor key. The sequence of these features was randomized, thereby also randomizing the difficulty level. Phrases for the rhythm subtest consisted of 4–11 beats, played at a tempo of 100 bpm on a wood block. “Different” trials contained a single rhythmic change. Rhythmic complexity was manipulated by including triplets in 21 trials, while the remaining 31 trials used only even beat subdivisions. Thirty-seven trials started on the downbeat, with the rest beginning later. The sequence of these features was also randomized across trials. The entire MET was completed within 20 minutes. Given that the study ran online without supervision, individual MET performance below the normed range of values on the melody and/or the rhythm subtest was used as a quality control measure and served as an exclusion criterion (see data pre-processing steps below).

In the final test, participants listened to looped test sentences and rated each sentence’s song-likeness after repetitions using the same scale as in the baseline test (1 labeled “clearly speech”, 8 "clearly song")^8,11^. At the end of the experiment, some basic demographic information was collected by means of a short questionnaire.

The study was approved by the Ethics Committee of the University of Konstanz (IRB statement 05/2021, dated 04/02/2021) and conducted in accordance with all relevant guidelines and regulations. All participants provided informed consent prior to their participation which lasted approximately 35–40 min.

### Data pre-processing and analyses

Data pre-processing and statistical analyses were conducted in Rstudio (running R-version 4.5.0)^68^. We used *lme4*,^69^ *lmerTest*,^70^ and *emmeans*^71^ libraries.

We first analyzed the MET data by counting the number of correct responses on each subtest and transforming the two counts into normed Z-scores^32^. The Z-score normalization reflects how a participant’s raw score compares to the distribution of scores in the large-scale reference sample of Canadian undergraduates (which included speakers of tonal as well as non-tonal languages) tested in previous work and is scaled to center around 100^32^. Using norming and normed data offered several methodological advantages. First, it improved comparability of obtained MET-scores with previous research and across the two MET-subtests used in the present study^32^. Second, it provided externally validated benchmarks for score distributions which served as an independent and more principled basis for quality control and participant exclusion decisions. This approach aligns with a growing methodological consensus cautioning against outlier removal based on sample-internal distribution properties^72^. Nine out of the 84 volunteers scored so low on one or both subtests of the MET that their result could not be assigned a normed score. Given that none of these participants disclosed amusia or related impairments and since the study was running online without supervision, we inferred that these participants did not fully pay attention to the tasks of the experiment and excluded their data from further analyses, by way of ensuring high quality of the overall dataset. The data of the remaining 75 participants are reported. To compare the MET performance of the present study’s sample, we ran correlations of the normed scores obtained in the rhythm vs. melody subtest of the MET and performed the Wilcoxon signed rank test with continuity correction to examine the difference between the two scores.

Song-likeness ratings collected in the two tasks (baseline, repetition) were z-score transformed. We first examined if the effect of repetition was observed in the Mandarin listeners of the present study by comparing song-like ratings they gave to test sentences before and after repetition in a linear mixed-effects model, and examined if phonological sonority (high- vs. low-sonority) influenced how song-like the test sentences were perceived. We then fitted two linear mixed-effects models to test if the hypothesized increase in song-likeness upon sentence repetitions was moderated by (1) individual rhythm perception ability or (2) melody perception ability. Two separate models were necessary because the two individual measures were correlated and could not be fit as predictors within the same model. All models further included two crossed random intercepts, participant and sentence. We allowed for a random slope of task over participant (which was retained in all models). Likelihood ratio tests were used to establish a factor’s contribution to the model fit^70^.

## Data Availability

The dataset collected and analysed for the present study is available from the corresponding author upon reasonable request.
